# MicroRNA: role in macrophage polarisation and colorectal cancer pathogenesis

**DOI:** 10.3389/fcell.2025.1619526

**Published:** 2025-07-23

**Authors:** Haihong Lin, Jun Zhou, Ying He, Yifan Zhu, Puwen Chen, Hongwei Yan, Junyun Huang, Ersheng Gong, Xiaoling Wang

**Affiliations:** ^1^ Department of Laboratory Medicine, The First Affiliated Hospital of Gannan Medical University, Ganzhou, China; ^2^ College of Medical Technology, Gannan Medical University, Ganzhou, China; ^3^ Department of Laboratory Medicine, Beijing Jishuitan Hospital Guizhou Hospital, Guiyang, China; ^4^ Department of Laboratory Medicine, Shantou Yuehui Orthopedic Hospital, Shantou, China; ^5^ The First School of Clinical Medicine, Gannan Medical University, Ganzhou, China; ^6^ Key Laboratory of Prevention and Treatment of Cardiovascular and Cerebrovascular Diseases (Ministry of Education), Gannan Medical University, Ganzhou, China; ^7^ School of Public Health and Health Management, Key Laboratory of Development and Utilization of Gannan Characteristic Food Function Component of Ganzhou, Gannan Medical University, Ganzhou, China

**Keywords:** microRNA, macrophages, colorectal cancer, extracellular vesicles, cancer

## Abstract

Colorectal cancer (CRC) represents a highly common gastrointestinal malignancy ranking among the top three most frequently diagnosed cancers in the digestive system. The disease’s high mortality rate makes treatment particularly difficult. As a result, thorough research into the cause and effective treatment of CRC is especially crucial. The macrophage’s remarkable functional flexibility, as a cell with strong immunological effects, allows it to demonstrate both anti-tumor and tumor-inducing activities. MicroRNAs (miRNAs), functioning as short non-protein-coding RNAs, mediate post-transcriptional regulation through mRNA destabilization and translational suppression, and they play a unique function in macrophage formation, polarization processes, and anti-inflammatory activity. Elucidating the crosstalk between miRNA-mediated gene regulation and macrophage functional polarization in CRC pathogenesis constitutes a critical research priority. We first provide a brief overview of the epidemiological of CRC, systematically summarising the origin of macrophages, their physiological functions, and their potential pathogenic mechanisms in colorectal carcinogenesis. Subsequently, we elaborated in depth on the critical role of miRNAs in regulating macrophage polarisation status. Ultimately, this paper comprehensively explores the mechanistic involvement of miRNA-macrophage interactions in CRC progression.

## 1 Introduction

Within the hierarchy of global cancer burden, Colorectal cancer (CRC) claims the third-highest incidence rate, yet escalates to the second predominant etiology of fatality among all oncologic pathologies (Baidoun et al., 2021). Statistics show that 7.7 to 8.5 percent of individuals in their 40s will develop CRC if they do not undergo a cancer screening program, and even more alarmingly, 3.2 to 3.4 percent of this group will eventually lose their lives to the disease (Sung et al., 2021; Sullivan et al., 2022). A rising incidence of CRC among individuals aged <50 years—now clinically designated as young-onset CRC (YO-CRC)—has been documented globally, with consistent patterns across geographically diverse regions and both genders. The development of colorectal cancer is influenced by multiple established risk determinants, including dietary habits, environmental factors, lifestyle, a history of intestinal diseases, and genetic factors (Matsuda et al., 2025). However, the implementation of comprehensive preventive measures—including maintaining a healthy lifestyle, obtaining regular cancer screenings, and seeking timely medical consultation—has been shown to effectively reduce the risk of CRC development. Given that CRC has emerged as a significant global health burden, the exploration of innovative therapeutic strategies and targeted treatment modalities has become increasingly imperative.

In the tumour microenvironment (TME), tumour-associated macrophages (TAMs) typically differentiate into two main subtypes: the classically activated M1 type and the alternatively activated M2 type (Li et al., 2021a). These two types of macrophages differ in surface indicators, functions, released cytokines, and roles in various illnesses, particularly tumors. Pro-inflammatory M1 macrophages activated by interferon-γ exhibit potent tumoricidal activity through direct cytotoxicity and immunostimulatory signaling, potentially serving as critical mediators of malignant cell elimination during initial tumor suppression (Wang et al., 2019). While M2-polarized TAMs are key drivers of oncogenesis and metastasis within the TME, their molecular signatures and immunosuppressive functions offer exploitable vulnerabilities for developing targeted therapies or prognostic indicators in cancer immunotherapy (Liu et al., 2024). MicroRNAs (miRNA) play key roles in macrophage development and regulate transcription factors in response to microenvironmental signals, which in turn control macrophage polarisation. miRNAs and macrophage presence will promote tumour cell EMT, accelerate tumour proliferation and invasion, promote angiogenesis, and enhance metastasis, suggesting that they have important aspects of their great potential (Zhang et al., 2023a). Finally, the ability of engineered macrophages to more effectively recognise, bind, and kill tumour cells or to modulate the immune microenvironment to inhibit tumour growth shows greater potential in tumour immunotherapy.

## 2 Overview of colorectal cancer

### 2.1 Epidemiology

Globally, colorectal neoplasms (CRC) hold the third position in cancer incidence, exhibiting an annual caseload of nearly 1.9 million individuals (Klimeck et al., 2023) (10 percent of all new cancer cases worldwide). The risk factors and histological classification of CRC are summarized in Tables 1, 2, respectively. For CRC patients with early localized lesions (i.e., stages I and II), the 5-year survival rate can approach 90%. However, the survival rate for patients with advanced CRC is less than 10 percent, mainly because advanced CRC spreads to distant organs (Li et al., 2021b). Epidemiological projections indicate that by 2040, the annual incidence of CRC is anticipated to rise by 63%, reaching 3.2 million new cases globally, while mortality rates are projected to escalate by 73%, amounting to 1.6 million deaths per year (Morgan et al., 2023). As a predominant malignancy within the gastrointestinal system, CRC presents a significant barrier to treatment efficacy because of its high lethality. As a result, it is critical to perform extensive research and exploration into the pathophysiology of CRC, as well as to identify effective therapy options.

**TABLE 1:** Risk factors for CRC.

Risk factors	Mechanisms of pathogenesis	References
Family-related	Lynch syndrome- germline mutation in 1 of the MMR genes Familial adenomatous polyposis- mutation in the *APC*gene	Peltomäki et al. (2023), Vasen et al. (2015)
Genders	Men are more likely to develop cancer than women. Androgens accelerate tumour growth by suppressing anti-tumour immune activity, reducing microbial diversity, and promoting the release of tumour-promoting factors from the nervous system	Rodríguez-Santiago et al. (2024)
Age	The incidence of the disease is concentrated in the age group of 50–69 years, and there is a trend towards a younger age group	Zhang et al. (2024a), Keum and Giovannucci (2019)
Catering	Higher Dietary Inflammatory Index scores are associated with pro-inflammatory potential, thereby increasing the risk of CRC.	Shivappa et al. (2017)
Alcohol consumption	Carcinogenic effects by interfering with DNA synthesis and repair, altering glutathione structure and function, and stimulating proliferation of the colonic mucosa	Thanikachalam and Khan (2019), ZHOU et al. (2022)
Obesity	Decreased lipocalin secretion promotes the emergence and development of CRC	Ye et al. (2020), Socol et al. (2022)
Hyperglycemia	Increased serum insulin and IGF-1 levels promote increased colorectal epithelial cells	Murphy et al. (2022)
Cigarette smoking	Chemical damage	Lim et al. (2024), Bai et al. (2022)

**TABLE 2:** Histological classification and features of serrated polyps in colorectal cancer.

Categorisation	Characterisation	References
Hyperplastic polyp	Uniform proliferation of the epithelium of the upper two-thirds of the crypt, forming small papillae, which extend into the lumen of the crypt, thus giving the surface of the lumen a jagged appearance	Wang et al. (2024a), Kim and Kang (2020)
Jagged non-tipped polyps	Similar in colour to the surrounding mucosa, with a flattened or non-tipped pattern	Kawasaki et al. (2016), Murakami et al. (2018)
Traditional serrated polyp	Twisted chorionic (filamentous) or tubular villous structures, in many cases with bulbous tips	Mccarthy et al. (2019)

### 2.2 Micro-driver genes and colorectal cancer

Chronic inflammation markedly elevates the mutation rate of essential driver genes by intensifying genomic instability in malignant cells (Vitale et al., 2019). Furthermore, micro-driver genes with diminished tumorigenic effects may potentially expedite carcinogenesis when they mutate simultaneously. Research indicates that during inflammation, pro-inflammatory cytokines (e.g., TNF-α) stimulate the generation of reactive oxygen species (ROS) and reactive nitrogen species (RNS) by activating many pro-oxidative enzymes, thus perpetuating oxidative stress and tissue injury (Burgos-Molina et al., 2024). Shimomura et al. discovered that TNF-α can induce senescence and activate senescence signaling pathways by augmenting the cellular plasticity of colonic epithelial cells, potentially explaining the mutations in senescence-related genes in inflammation-associated colonic tumors resulting from selective pressure (Shimomura et al., 2023). Multiple cumulative micro-driver genes can combine to form major drivers with sufficient influence to alter cell function and affect patient prognosis (Badr et al., 2022). Campos Segura et al. discovered five genes potentially functioning as micro-driver genes for colorectal cancer using computational analysis: *DOCK3, FN1, PAPPA2, DNAH11*, and *FBN2*, which are linked to mechanisms of cancer cell invasion and metastasis (Campos Segura et al., 2023). Moreover, patients possessing these gene mutations exhibit reduced survival rates in comparison to those without mutations (Campos Segura et al., 2023). Identifying micro-driver genes in CRC can facilitate the discovery of novel biomarkers, evaluate patient prognosis, and establish a foundation for the development of individualized treatment strategies.

## 3 Macrophages and their regulatory role in colorectal cancer

### 3.1 Phenotype and function of macrophages

**FIGURE 1:**
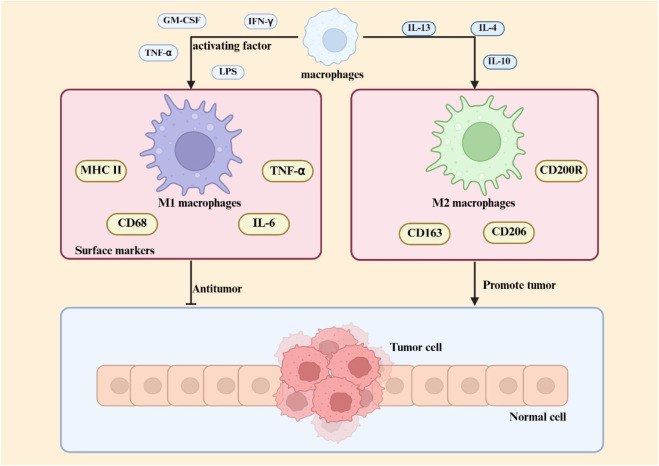
Macrophage polarization in the TME Within the TME, macrophage polarization is regulated by multiple factors, including cytokines, metabolic signals, and transcriptional pathways. M1 macrophage polarization is primarily driven by Th1-type cytokines (e.g., IFN-γ) and pathogen-associated molecules (e.g., LPS), with molecular mechanisms involving the STAT1 and NF-κB signaling pathways. M2 macrophage differentiation is induced by Th2-type cytokines (e.g., IL-4, IL-13) and immunosuppressive factors (e.g., IL-10), and its core regulatory mechanisms rely on multiple pathways, including STAT3/STAT6 signaling.

The classification of macrophages is a contentious issue, influenced by various factors that result in distinct phenotypes and activation states, including growth factors, receptors, signaling pathways, and transcription factors (Yuan et al., 2023a). Macrophages can be categorized based on their function and activation into two subtypes: traditionally activated M1 macrophages and alternatively activated M2 macrophages. Certain cytokines, including granulocyte-macrophage colony-stimulating factor, tumor necrosis factor α, and interferon, either independently or in conjunction with lipopolysaccharides, effectively activate macrophages and induce their transformation into the M1 phenotype (Chen and Zhang, 2017). M1 macrophages are essential to the immune system, especially in defense against intracellular infections (Atri et al., 2018). Activated by inflammatory signals, M1 macrophages upregulate inducible nitric oxide synthase to metabolize L-arginine into nitric oxide, a key antimicrobial agent critical for pathogen clearance (Martinez et al., 2008). These cells exhibit elevated surface expression of MHC class II molecules and CD68 markers, while demonstrating enhanced secretion of pro-inflammatory mediators including interleukin-6 and tumor necrosis factor-alpha (Vago et al., 2020). M2 macrophages exhibit functions extending beyond antimicrobial defense, including clearance of apoptotic cells, mitigation of inflammatory processes, and promotion of tissue repair. (Sharifiaghdam et al., 2022; Hu et al., 2021). These cells are characterized by specific surface markers (CD200R, CD206, CD163) and functional molecules (Arg-1, STAT-3), alongside secretion of anti-inflammatory cytokine IL-10 (Bhattacharya and Aggarwal, 2019) Current classification divides M2 macrophages into three subtypes (M2a, M2b, M2c) (Bhattacharya and Aggarwal, 2019), with distinctions arising from variations in surface receptor profiles, cytokine secretion patterns, and specialized biological functions. Figure 1 shows the differentiation and function of macrophages.

Tumour-associated macrophages, a crucial element of the TME, are intricately linked to cancer genesis, progression, and metastasis (Zhang et al., 2024b). M1 macrophages demonstrate anti-tumor properties by facilitating phagocytosis and antibody-dependent cell-mediated cytotoxicity to eliminate tumor cells (Basak et al., 2023). M2 macrophages can suppress T cell-mediated anti-tumor immune responses, enhance tumor angiogenesis, and increase tumor development and metastasis (Liu and Cao, 2015). In the initial phases of cancer, M1 macrophages are predominant, while in the subsequent stages, there is a gradual increase in M2 macrophages (Mantovani et al., 2002). The phenotype and characteristics of macrophages are summarized in Table 3.

**TABLE 3:** Phenotypes and characteristics of macrophages.

Characteristics	M1 macrophage	M2 macrophage	References
Functionality	Pro-inflammatory, anti-tumor, anti-pathogen	Anti-inflammatory, tumor promotion, tissue repair	Li et al. (2023a), Mehla and Singh (2019)
Metabolism	Dependent on glycolysis, producing ROS and NO	Dependence on oxidative phosphorylation and fatty acid oxidation	Mouton et al. (2020), Li et al. (2024a)
Subtype	undefined	M2a, M2b, and M2c	Zhang and Sioud (2023)
Surface marker	MHC II, CD68, IL-6, and TNF-α	CD200R, CD206, CD163, Arg-1, STAT-3, and IL-10	Vago et al. (2020), Bhattacharya and Aggarwal (2019)
Related diseases	Atherosclerosis, acute infection	Tumors, allergic diseases	Wang et al. (2019), Lv et al. (2024), Sharma et al. (2018)
Signal pathway	STAT1, NF-κB	STAT6, STAT3	Wu et al. (2021), Wang et al. (2022a), Deng et al. (2024)
Macrophage activating factor	IFN-γ, TNF-α, LPS, and GM-CSF	IL-4, IL-10, IL-13, and glucocorticoids	Chen and Zhang (2017), Yunna et al. (2020), Shrivastava and Shukla (2019)

### 3.2 Macrophages play a role in colorectal cancer

The composition of the TME is well acknowledged as a catalyst for solid tumor development, significantly influencing tumor progression, size, evolution, and responsiveness to diverse therapies. In this microenvironment, infiltrating immune cells are pivotal since they can either facilitate tumor control or contribute negatively to malignant tumor progression (Mola et al., 2020). Macrophages contribute to the formation of inflammatory tumor microenvironments during the development of inflammatory bowel disease (IBD) and CRC. Genetic disruption of the Stat3 gene in macrophages impairs IL-10-mediated anti-inflammatory signaling, thereby triggering persistent intestinal inflammation and promoting the development of neoplastic lesions (Deng et al., 2010).

Several studies on IBD mouse models have pointed out that during the onset of colitis, pro-inflammatory macrophages usually accumulate in the colonic mucosa (Lu et al., 2024). A study demonstrated through experiments in a T-cell-mediated mouse model of colitis that Pro-inflammatory macrophages quickly became the main macrophage population in the mesenteric lymph nodes of the colon. Just 12 h post T-cell transplantation, with this proportion remaining significantly elevated 3 weeks later, constituting over half of the total colonic macrophages (Tamoutounour et al., 2012). Yes-associated protein (YAP) has been demonstrated to be a significant promoter of tumorigenesis (Wang et al., 2016). Xin Zhou et al. have shown that YAP intensifies inflammatory bowel disease through the modulation of M1/M2 macrophage polarization and the maintenance of gut microbial equilibrium (Zho et al., 2019). The S1PR signaling pathway is critically involved in sustaining macrophage-driven chronic inflammatory responses (Weigert et al., 2019). Activation of the *S1PR2/RhoA/ROCK1* axis aggravates inflammatory bowel disease through dual mechanisms: disrupting intestinal vascular endothelial barrier integrity and enhancing pro-inflammatory M1 macrophage polarization, as evidenced by research published in Biochemical Pharmacology (Wang et al., 2022b). Phagocytic removal of apoptotic epithelial cells by intestinal macrophages is essential for maintaining epithelial integrity. Experimental evidence demonstrates that targeted depletion of these macrophages leads to impaired clearance of cellular debris, triggering epithelial barrier disruption and subsequent colonic inflammation (Huynh et al., 2013). *Gpr84* is a G protein-coupled receptor expressed in several myeloid cells, including macrophages. Zhang Qing et al. discovered that *Gpr84* expression was markedly elevated in the inflamed colonic tissues of individuals with active ulcerative colitis (UC) and in animals with dextran sulfate sodium (DSS)-induced colitis. *Gpr84* expression was markedly elevated in colonic tissues, and GPR84 signaling promoted intestinal mucosal inflammation through enhanced *Nlrp3* inflammasome activation in macrophages (Zhang et al., 2022). The progression of IBD-related colorectal cancer is attributable to the cumulative impact of persistent intestinal inflammation. Chronic inflammation induces epithelial growth, advancing from heterogeneous hyperplasia to adenocarcinoma. This is a multi-stage, multifactorial, multigenic process (Rogler, 2014; Wijnands et al., 2021).

Following tumor formation, M1 has an anti-tumor effect by direct tumor cytotoxic mechanisms or by facilitating the activity of CD8 cytotoxic T cells and NK cells to eliminate tumor cells during the advanced stages of colorectal cancer progression. M2 facilitates tumor immune evasion and accelerates tumor growth, particularly invasion and metastasis (Zhang et al., 2023b). Research conducted at the University of Verona demonstrated that M2 polarized macrophages can synthesize IL13 and CCL17, which contribute to modifications in glycosylation in epithelial cells, hence facilitating ulcerative colitis and colon cancer (Bronte, 2020). Shunyi Wang et al. demonstrated that macrophage-specific Act1 downregulation induces STAT3 activation, driving adenoma-adenocarcinoma progression through dual pathways involving the CXCL9/10-CXCR3 and PD-1/PD-L1 axes within CD8^+^ T cells (Wang et al., 2023). Qing Liu et al. demonstrated that Wnt5a induces IL-10 secretion in macrophages, which acts through autocrine signaling to drive their M2 polarization, and these polarized M2 macrophages consequently enhance colorectal cancer cell proliferation, migration, and invasion (Liu et al., 2020). A study published in Cancer Research demonstrated that M2 macrophage-derived extracellular vesicles are abundant in miRNAs that penetrate colorectal cancer cells and associate with the coding sequence of BRG1, a recognized pivotal promoter of colorectal cancer metastasis, thereby expediting the invasion and metastasis of colorectal cancer (Lan et al., 2019).

## 4 miRNA regulation of macrophages

### 4.1 Overview of miRNAs

A class of tiny non-coding RNAs known as miRNAs is involved in the post-transcriptional control of gene expression. RNA polymerase II converts genes containing miRNA information into longer primary transcripts, or pri-miRNAs (Ferragut Cardoso et al., 2021). RNase endonuclease III and double-stranded RNA-binding proteins break the pri-miRNA into precursor miRNA. The transporter proteins Exportin-5 and Ran-GTP move pre-miRNA from the nucleus to the cytoplasm. The enzyme Dicer further cleaves pre-miRNAs in the cytoplasm to create mature double-stranded miRNA duplexes (Krol et al., 2010). By base-pairing to the 3′end of the untranslated region of their particular target mRNA, mature miRNAs destabilize and translationally silence the mRNA, hence inhibiting protein expression (Fabian et al., 2010). A miRNA can often control several mRNAs simultaneously, and several miRNAs can co-regulate one mRNA (Tajbakhsh et al., 2019). Cardiovascular, neurological, metabolic, and infectious disorders are just a few of the many illnesses whose origin and progression are strongly linked to miRNAs (Paul et al., 2018). Additionally, there is a strong correlation between miRNAs and cancer. Numerous miRNAs exhibit abnormal expression in human malignancies and influence the processes of cancer cell differentiation, proliferation, and apoptosis, hence serving as either tumor promoters or suppressors. The degree of malignancy, metastasis, and prognosis of cancer are all correlated with abnormally expressed miRNAs, which may be used as biomarkers for cancer diagnosis (He et al., 2020). As a result, miRNAs are now frequently the subject of cancer research and novel treatment strategies.

### 4.2 miRNA regulation of macrophage development

**FIGURE 2:**
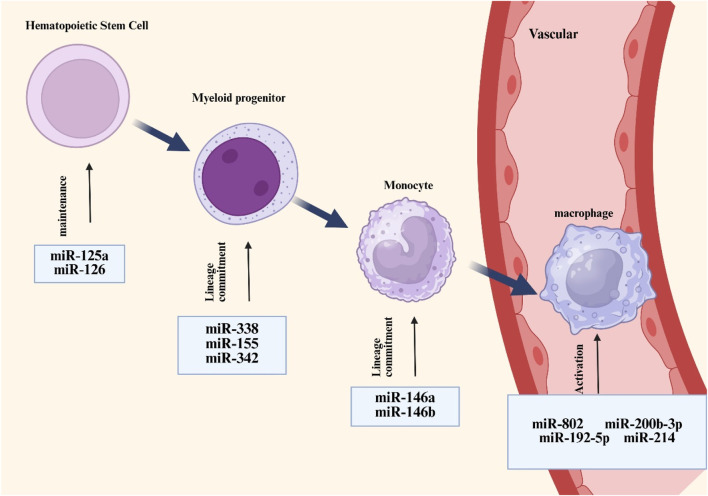
Overview of miRNAs associated with macrophage development and activation. MiRNAs such as miR-125a and miR-126 are responsible for the maintenance of hemotopoietic stem cells, whereas other types of miRNAs such as miR-338 and miR-155 regulate the differentiation of hemotopoietic cells towards mononuclear phagocytic cell lineages (which are at homeostasis) and are involved in controlling macrophage activation processes.

Monocytes originate predominantly from hematopoietic stem cells in the bone marrow. In the bone marrow, these stem cells go through a number of differentiation processes to become monocytes, which are then transported by blood flow to other bodily regions. After migrating into tissues, monocytes continue to differentiate, grow in size, proliferate mitochondria and endoplasmic reticulum, improve phagocytosis, and eventually mature into macrophages (Rigamonti et al., 2023). According to preliminary research, arsenic resistance protein 2 (ARS2) mediates the important role that miRNAs play in controlling the function of hematopoietic stem cells (Gruber et al., 2009). Ars2 is a crucial protein that contributes to the pri-miRNA microprocessor complex process by transporting miRNA transcripts (Yuan et al., 2023b). MiRNAs are essential for preserving normal hematopoietic stem cell activity, as seen by the notable bone marrow failure phenotype seen in ARS2-deficient mouse models (Gruber et al., 2009). miR-126, expressed in hematopoietic stem cells, suppresses cell cycle progression and hematopoietic activity by modulating the PI3K/AKT pathway, thereby restricting the responsiveness to extrinsic signals such as stem cell factors (Lechman et al., 2012). In addition, colony-stimulating factor-1 (CSF-1) interacts with its receptor CSF-1R to promote the differentiation and maturation of monocyte lineages. The expression of CSF-1R is regulated by Runt-related transcription factor-1 (RUNX1, also known as AML1), while miRNA 17-5p can regulate monocyte generation by targeting AML1 (Fontana et al., 2007). In pluripotent progenitor cells, CCAAT enhancer binding protein α (C/EBPα) is upregulated, and its activity is controlled by miR-182 and miR-34. c/EBPα directly upregulates the expression of miR-223 and miR-34, which are co-formers of myeloid progenitor cells (which eventually differentiate into macrophages and dendritic cells), which is critical (Stavast et al., 2018). The differentiation of myeloid lineages is controlled by miRNAs through mechanisms functionally coupled with PU.1 activity. Specifically, PU.1 transcriptionally activates miR-338, miR-155, miR-342, and miR-146a, collectively promoting myeloid cell maturation through their differentiation-supportive functions. (Ghani et al., 2011). In addition, researchers have observed that miRNAs are elevated in haematopoietic stem cells and upregulated during senescence. For instance, miRNA-132 regulates senescent hematopoietic stem cells by acting on the transcription factor FOXO3, a well-known senescence-associated gene (Mehta et al., 2015). Figure 2 shows the differentiation of hematopoietic stem cells into macrophages.

### 4.3 miRNA regulation of macrophage polarisation

#### 4.3.1 Macrophage polarisation

As multipurpose sentinels of the innate immune system, macrophages are essential for tissue homeostasis, disease etiology, and host defense. The M1 or M2 phenotype is the result of macrophages differentiating from their primitive monocyte state when they detect external stimuli through their membrane receptors. Intracellular signaling cascades are transduced during this process, which ultimately affects the expression of macrophage genes. Changes in protein levels that are triggered as a result help macrophages adapt functionally and establish particular phenotypes. The activation state of macrophages is influenced by metabolic reprogramming, nuclear receptors, STAT proteins, Toll-like receptors (TLR), and epigenetic changes (Yan et al., 2020). The interaction of TLRs with their ligands activates the recruitment of adaptor proteins, including myeloid differentiation primary response 88 (MyD88), which commences downstream signaling cascades (Onyishi et al., 2023). These cascades result in the activation of transcription factors that subsequently promote the expression of pro-inflammatory genes and define the M1 macrophage phenotype. STAT proteins are a category of proteins that are integral to cellular signal transduction. They can be stimulated by various cytokines and growth factors, regulating gene transcription via nuclear translocation. In macrophages, the activation of STAT proteins influences their activation status and functionality. The activation of STAT1 is typically linked to the polarization of M1-type macrophages, while the activation of STAT6 is connected with the polarization of M2-type macrophages (Li et al., 2020). Epigenetic factors can modulate macrophage polarization through the mediation of DNA methylation and histone changes, facilitating the transition from a reparative to an inflammatory M2 phenotype and influencing the expression of pro- and anti-inflammatory genes in macrophages (Eriksson Ström et al., 2022; Zhang et al., 2024c). Moreover, miRNAs, as pivotal epigenetic regulatory molecules, are integral to the intricate process of macrophage polarization. MiRNAs meticulously modulate the gene expression network linked to macrophage polarization, hence affecting the trajectory of macrophage development towards either pro-inflammatory M1-type or anti-inflammatory M2-type. This miRNA-mediated regulation system offers new insights into macrophage functional diversity and its role in inflammatory responses, tissue healing, and disease progression.

#### 4.3.2 miRNA and macrophage polarisation

MiRNAs modulate the polarization state of macrophages by targeting and regulating essential genes related to polarization, such as cytokines, transcription factors, and other signaling pathways. Dai’s team discovered that miR-122 was markedly increased in liver metastasis of colorectal cancer. The overexpression of miR-122 enhanced the proliferation and migration of MC38 cells while suppressing the expression of *NEGR1*. *NEGR1* knockdown activated the PI3K/AKT pathway, facilitated M2 macrophage polarization and IL-10 secretion, and expedited liver metastasis of colorectal cancer *in vivo* (Dai et al., 2024). Targeting NEGR1 knockdown is a promising approach for the prediction and treatment of colorectal cancer liver metastases (Dai et al., 2024). This discovery elucidates a novel interaction mechanism between neoplastic cells and immune cells within the tumor microenvironment, providing fresh insights into cancer research. Zhang et al. discovered that miR-192-5p effectively targeted interleukin-1 receptor-associated kinase 1 (IRAK1), prompting myocardial infiltrating macrophages to predominantly adopt the M2 phenotype, thereby successfully safeguarding mice from the fatal viral myocarditis induced by coxsackievirus B3 (CVB3) (Zhang et al., 2020).

Extracellular vesicles (EVs) are diminutive vesicles secreted by many cells that can significantly contribute to intercellular communication. EVs contain many miRNA that significantly influence the phenotypic of receiving cells (Mulcahy et al., 2014). Table 4 summarizes the biological mechanisms by which miRNAs carried by EVs influence macrophage polarization. Zhang et al. demonstrated that miRNA-363 and miRNA-709, packaged within CD63^+^ graft macrophage-derived evs, drove splenic M1 polarization in recipients via the Fcho2/Notch1 axis, establishing that targeting vesicular transport from graft-associated macrophages represents a viable therapeutic strategy against rejection (Zheng et al., 2025). Research conducted by Xu et al. has shown that extracellular vesicles enriched with miR-200b-3p, secreted by hepatocellular carcinoma (HCC) cells, facilitated macrophage proliferation and polarization through the modulation of cytokine production and the activation of the JAK/STAT signaling pathway, hence promoting metastasis of hepatocellular carcinoma (Xu et al., 2023). EVs originating from laminar shear stress (LSS-EVs) are essential for sustaining vascular homeostasis. Li et al. revealed that LSS-EVs were abundant in miR-34c-5p and reprogrammed macrophages by targeting the TGF-β-Smad3 signaling pathway, facilitating the transition from the macrophage M1 phenotype to the M2 phenotype (Li et al., 2025). This discovery is significant for elucidating the protective mechanisms of atherosclerosis. There is growing evidence that TME-associated macrophages have a mixed phenotype that secretes pro-inflammatory cytokines and immunosuppressive molecules (Lu et al., 2024). This mixed phenotype is controlled by the inositol-requiring enzyme 1 (IRE1)-X-box binding protein 1 (XBP1) axis, making Xbp1 an important target (Batista et al., 2020). Gonzalo Almanza et al. used engineered EVs as delivery vectors carrying miR-214 complementary to the 3′-UTR of Xbp1 to target and regulate Xbp1 expression in macrophages, thereby affecting macrophage phenotype (Almanza et al., 2024). Furthermore, EVs generated by glioblastoma (GBM) cells in hypoxic environments can be internalized by macrophages. Stoyan Tankov et al. discovered that miR-25/93-containing extracellular vesicles generated by hypoxia-induced glioblastoma cells suppress the cGAS-STING pathway and M1-related gene expression in macrophages, hence compromising the anti-tumor efficacy of these macrophages (Tankov et al., 2024). In comparison to normoxic glioma extracellular vesicles, Qian et al. also discovered that hypoxic glioma extracellular vesicles dramatically increased M2 macrophage polarization. They also showed that this process was achieved by delivering miR-1246, which activates the STAT3 pathway (Qian et al., 2020). Ge et al. identified miRNAs abundant in extracellular vesicles obtained from mice hearts, lungs, livers, and kidneys, specifically miR-148a-3p, miR-1a-3p, and miR-143-3p. MiRNAs abundant in these EVs mitigated the inflammatory responses of LPS-stimulated macrophages via many pathways, including STAT3, P65, and SAPK/JNK (Ge et al., 2024). An investigation published in International Immunopharmacology demonstrated that serum extracellular vesicle-derived miR-146a-3p facilitated macrophage M2 polarization in allergic rhinitis (AR) by targeting VAV3 through the PI3K/AKT/mTOR signaling pathway. This finding indicates that miR-146a-3p and VAV3 may serve as prospective targets for the formulation of novel therapeutic methods for AR (Xia et al., 2023). Zhou et al. revealed that extracellular vesicles produced from tumor cells enriched with miR-184-3p were internalized by macrophages, leading to the inhibition of the JNK signaling cascade through the targeting of EGR1, which subsequently induced M2 polarization in macrophages. Moreover, the tumor suppressor miR-184-3p was identified as being actively incorporated into extracellular vesicles following its interaction with heterogeneous nuclear ribonucleoprotein A2B1 (hnRNPA2B1), thereby facilitating tumor cell proliferation and metastasis; obstructing its secretion significantly curtailed tumor growth and metastasis (Zhou et al., 2023).

**TABLE 4:** Biological mechanisms by which miRNAs carried by EVs influence macrophage polarization.

Sources of EVs	Carried miRNA	Target/Mechanism	Polarization	References
CD63^+^graft macrophages	miRNA-363,miRNA-709	Fcho2/Notch1	Enhanced M1 polarization	Zheng et al. (2025)
Hepatocellular carcinoma cells	miR-200b-3p	JAK/STAT	Enhanced M2 polarization	Xu et al. (2023)
Laminar shear stress in blood vessels	miR-34c-5p	TGF-β-Smad3	Promoting polarization from M1 phenotype to M2 phenotype	Li et al. (2025)
Mouse B cells	miR-214	Regulation of Xbp1 expression in macrophages	Reduce mixed phenotypes	Almanza et al. (2024)
Hypoxic glioblastoma cells	miR-25/93	cGAS-STING	Lower M1 polarization	Tankov et al. (2024)
Hypoxic glioblastoma cells	miR-1246	STAT3	Enhanced M2 polarization	Qian et al. (2020)
Mouse organs	miR-148a-3p, miR-1a-3p, and miR-143-3p	STAT3, P65, and SAPK/JNK	Lower M1 polarization	Ge et al. (2024)
Serum	miR-146a-3p	PI3K/AKT/mTOR	Enhanced M2 polarization	Xia et al. (2023)
Breast cancer cells	miR-184-3p	Targeted EGR1 inhibition of JNK	Enhanced M2 polarization	Zhou et al. (2023)

## 5 miRNAs and macrophages in colorectal cancer

### 5.1 miRNAs and macrophages in colorectal cancer

Currently, it is widely believed that TAMs play a series of primarily harmful roles in cancer progression. Local chronic inflammation, especially low-grade inflammation, in which macrophages continuously produce low levels of pro-inflammatory cytokines and reactive oxygen species (ROS), can promote tumorigenesis by promoting genomic instability in malignant cells while interfering with the ability of resident macrophages to distinguish between healthy somatic cells and transformed cells (Vitale et al., 2019). As initial cancer cell clones proliferate densely, macrophages start to perceive the tumor as healthy tissue and adhere to the directives of cancer cells. In this co-evolving cancer ecosystem, tumor-associated macrophages facilitate tumor proliferation and circumvent several immune defenses (Kloosterman and Akkari, 2023). Tumor cells themselves can also evade immune surveillance by expressing higher levels of immune checkpoint molecules such as PD-L1 (Gou et al., 2020). TAMs results in the secretion of various cytokines that facilitate cancer progression by inducing epithelial-mesenchymal transition (EMT), promoting angiogenesis, initiating metabolic reprogramming, enhancing multidrug resistance, and imparting cancer stem cell traits, among other effects (Wang et al., 2021). In addition, as shown in Table 5, miRNAs influence disease progression by regulating macrophage polarization in various diseases.

**TABLE 5:** miRNAs promote macrophage polarisation in various diseases.

Illnesses	MiRNA	Expression of miRNA	Regulation of macrophage phenotype	References
Systemic lupus erythematosus	miR-122-5p	High expression	Promotion of M1	Ji et al. (2024)
Periodontitis with T2DM	miR-155	High expression	Promotion of M1	Mathews et al. (2024)
Renal ischaemia-reperfusion injury	miR-21	Low expression	Promotion of M2	Wang et al. (2024b)
Acute pancreatitis	miR-181a-5p	High expression	Promotion of M2	Li et al. (2024b)
Adipose tissue inflammation	miRNA-802	High expression	Promotion of M1	Yang et al. (2024a)
Xerophthalmia	miR-214-3p	High expression	Inhibition of M1	Zhao et al. (2024)
Sepsis	miR-31	Low expression	Inhibition of M2	Zhou et al. (2024)
Kidney damage and fibrosis	miR-92a-3p	High expression	Promotion of M1	Xu et al. (2024)
Unilateral ureteral occlusion	miR-126	High expression	Promotion of M1	Luo et al. (2024)
Chronic heart failure	miR-21-3p	High expression	Promotion of M1	Huang et al. (2024)
Glioma cell	miR-25-3p	High expression	Promotion of M2	Xue et al. (2024)
Squamous cell carcinoma of the cervix	miR-204-5p	High expression	Promotion of M2	Chen et al. (2024)
Osteosarcoma	miR-487a	High expression	Promotion of M2	Wang et al. (2024c)

EMT is the process by which cells lose their epithelial qualities and gain mesenchymal characteristics, which is critical in tumor growth, metastasis, and medication resistance (Pastushenko and Blanpain, 2019). TAMs promote cancer progression through EMT by activating NF-κB, inflammatory cytokines, and growth factors, including IL-6 (Hu et al., 2024; Song et al., 2017). Tumour-derived EVs containing miR-106b-5p have been demonstrated to stimulate the PI3K/AKT/mTOR signaling cascade by suppressing PDCD4 expression, so prompting macrophages to transition to an M2-polarised state. The activated macrophages subsequently facilitate the EMT of tumor cells, increasing their invasiveness, fostering the development of circulating tumor cells (CTCs), and finally expediting the metastatic progression of CRC to the liver and lungs (Yang et al., 2021). Chen et al. discovered that macrophages adjacent to CRC cells, upon targeted modulation, produce IL-6 to influence the EMT process, thereby augmenting the migratory and invasive capabilities of CRC cells. IL-6 secreted by TAMs promotes the JAK2/STAT3 signaling pathway, subsequently activating the STAT3 transcription factor that suppresses the production of the tumor suppressor miR-506-3p in CRC cells. The downregulation of miR-506-3p in colorectal cancer cells results in elevated FoxQ1 expression, whereas the upregulation of FoxQ1 enhances the synthesis of the chemokine CCL2, therefore attracting additional macrophages. The cyclic process can be obstructed by limiting the synthesis of CCL2 or IL-6, thereby diminishing macrophage migration and CTC-mediated metastasis, respectively (Wei et al., 2019). Wang et al. revealed that CXCR4 upregulation in CRC cells mediates Evs-dependent transfer of specific miRNAs (miR-25-3p, miR-130b-3p, miR-425-5p) to macrophages, driving their M2 polarization through PTEN suppression-mediated PI3K/Akt pathway activation. This polarization mechanism subsequently stimulated the release of EMT and vascular endothelial growth factor (VEGF), hence augmenting the metastatic potential of CRC. Their clinical analysis revealed that extracellular vesicular miRNAs (notably miR-25-3p, miR-130b-3p, and miR-425-5p) extracted from the serum of colorectal cancer patients are anticipated to serve as innovative non-invasive biomarkers for forecasting cancer development and metastasis (Wang et al., 2020).

Abnormal proliferation, invasion, and other functions collectively define the malignant biological behavior of tumor cells, which significantly contribute to the challenges in curing tumors and their tendency to recur. In the TME, TAMs and tumor cells engage with one another via mediators such as cytokines to facilitate cellular proliferation (Li et al., 2023b). Bao et al. established that tumor-derived γ-aminobutyric acid (GABA) drives macrophage M2 polarization in CRC. This polarization enhancement was attributed to the activation of the MAPK signaling pathway in macrophages by miR-223-3p molecules contained in EVs released by the tumor. Moreover, these M2-polarized macrophages significantly enhance the proliferation and migratory capacity of CRC cells through the secretion of IL-17 cytokines (Bao et al., 2023). The function of miR-1827, which is transported via EVs of human umbilical cord-derived mesenchymal stem cells, in colorectal cancer is thoroughly examined in a paper published by APOPTOSIS. Experimental data revealed that EVs suppressed SUCNR1 expression, effectively impairing M2 macrophage polarization. This suppression subsequently curbed CRC cell proliferation, migratory capacity, and invasive potential while markedly diminishing hepatic metastasis of CRC. This discovery offers a fresh viewpoint on how extracellular vesicles and miRNAs function in tumors (Chen et al., 2023a). Zhang et al. demonstrated significant elevation of miR-183-5p in M2-polarized tumor-associated macrophage-derived EVs, playing a pivotal role in CRC pathogenesis. This miRNA facilitates AKT/NF-κB pathway activation through THEM4 targeting, driving malignant progression via enhanced tumor cell proliferation, invasion, and metastatic dissemination (Zhang et al., 2021a).

Metastatic progression accounts for the predominant majority (approximately 90%) of cancer-related mortality. TAMs mediate tumor progression through multicellular crosstalk. These immune effector cells secrete proteolytic enzymes including matrix metalloproteinases (MMPs) to enzymatically disrupt extracellular matrix integrity, thereby enabling tumor cell dissemination (Meza-Morales et al., 2024). To facilitate metastasis, TAMs enhance the release of immunosuppressive cytokines, including IL-1ra, by augmenting tumor stemness (Wang et al., 2018). Liang et al. discovered that the ratio of M2-type macrophages was markedly elevated in colorectal cancer patients with liver metastases relative to those without liver metastases. The underlying mechanism of this phenomenon is that highly metastatic CRC cells secrete extracellular vesicles enriched with elevated levels of miR-106a-5p. These EVs drive M2 macrophage polarization through SOCS6 suppression and JAK2/STAT3 pathway activation. In clinical practice, increased levels of miR-106a-5p in extracellular vesicles in plasma are frequently linked to liver metastases and an unfavorable prognosis in colorectal cancer patients (Liang et al., 2024). Gut Microbes research demonstrated elevated *Fusobacterium* nucleatum in both fecal and tumor samples from CRC patients, showing stage IV versus stage I enrichment among metastatic cases. *Fusobacterium* nucleatum facilitates macrophage infiltration and generates M2 polarization via CCL20 activation while modulating miR-1322 expression, hence augmenting CRC metastasis through the miR-1322/CCL20 axis (Xu et al., 2021). Zhao et al. identified that miR-934, present in EVs derived from colon cancer, functions as a crucial regulatory molecule by releasing cytokines like CXCL13, which then activates the PI3K/AKT signaling pathway. This activation process enhances the M2-type polarization of macrophages at the molecular level, hence expediting the metastatic progression of CRC to the liver. This offers a novel perspective for comprehending the molecular mechanisms behind liver metastasis in colon cancer and may serve as a foundation for the formulation of innovative therapeutic methods aimed at this malignant metastatic process (Zhao et al., 2020).

**FIGURE 3:**
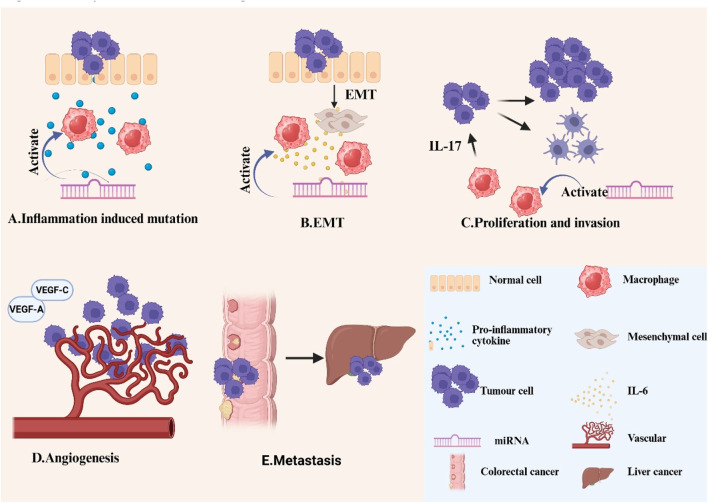
Role of macrophages and miRNAs in CRC **(A)**Inflammation induced mutation: The continued production of low levels of pro-inflammatory cytokines and reactive oxygen species by TAMS leads to genomic instability in malignant cells. **(B)**EMT: TAMs accelerate the CRC process by producing inflammatory cytokines and growth factors, such as IL-6, through processes such as EMT. **(C)**Proliferation and invasion: TAMs and tumour cells interact with each other via cytokines such as IL-17 to enhance tumour cell proliferation and invasion. **(D)**Tumour angiogenesis: TAMs promote angiogenesis mainly through the production of various pro-angiogenic factors (VEGFA, VEGFC, etc.). **(E)**Colorectal cancer liver metastasis: To promote metastasis, TAMs upregulate the secretion of immunosuppressive cytokines such as IL-1ra by increasing tumour stemness. In addition, miRNAs can promote macrophage polarization towards the M2 phenotype and suppress anti-tumor immune responses within the tumor microenvironment, hence facilitating tumor growth and progression.

TAMs are recognized for their role in regulating and facilitating angiogenesis. In murine cancer models, the reduction of TAMs impedes tumor angiogenesis, while the reconstitution of TAMs facilitates angiogenesis (Lin et al., 2006). Hypoxia inside the tumor microenvironment emulates the metabolic adaptability and pro-angiogenic characteristics of TAMs.TAMs facilitate angiogenesis primarily by producing several pro-angiogenic factors (such as VEGFA and VEGFC), which enhance tumor growth by promoting endothelial cell proliferation, causing sprouting, tube formation, and neointimal maturation (Shaw et al., 2024). A study published in CANCER LETTERS indicates that CRC cells that overexpress CXCR4 utilize extracellular vesicles to transport various miRNAs (such as miR-25-3p, miR-130b-3p, and miR-425-5p) to macrophages, thereby activating the PTEN/PI3K/Akt signaling pathway and prompting macrophages to polarize towards the M2 phenotype. Consequently, these polarized M2-type macrophages markedly augmented the metastatic capability of colorectal cancer by facilitating the EMT and elevating VEGF secretion (Wang et al., 2020). This research presents a novel therapeutic approach utilizing extracellular vesicle-encapsulated miRNA to target tumor-associated macrophage polarization in order to mitigate cancer spread. Figure 3 shows the progression of cancer.

### 5.2 Analysis of ceRNA in the colon, rectum, and rectosigmoid junction

The selection of treatment for colorectal cancer necessitates thorough evaluation of the tumor’s anatomical site (colon cancer, rectal cancer, or cancer at the rectosigmoid junction) and its molecular attributes. Current research indicates that the regulatory interaction between miRNA and mRNA/lncRNA differs markedly across various regions of the digestive system. Qi et al. revealed the functions of KCNQ1OT1 and SNHG1 in colorectal cancer through different ceRNA mechanisms based on the long non-coding RNA (lncRNA)-related competitive non-coding RNA (ceRNA) network (LceNET) (Qi et al., 2020). For example, the KCNQ1OT1/miR-484/ANKRD36 axis is involved in the development of colon cancer, while the KCNQ1OT1/miR-181a-5p/PCGF2 axis is associated with metastasis in rectal cancer; The SNHG1/miR-484/ORC6 axis plays a role in colon cancer, while the SNHG1/miR-423-5p/EZH2 and SNHG1/let-7b-5p/ATP6V1F axes are involved in the development and metastasis of rectal cancer, respectively (Qi et al., 2020). A study published in *Frontiers in Oncology* revealed many site-specific prognostic biomarkers for colorectal cancer: hsa-miR-1271-5p, NRG1, hsa-miR-130a-3p, SNHG16, and hsa-miR-495-3p in the colon; E2F8 in the rectum; and DMD and hsa-miR-130b-3p in the rectosigmoid junction (Vieira et al., 2021). Neoplasms at the intersection of the rectum and sigmoid colon are rather prevalent; however, detailed data on these tumors is limited due to their frequent categorization as either colon or rectal cancer. Zhang et al. discovered that the upregulated KCNQ1OT1 may compete with five significant DEmiRNAs to modulate the expression of target genes. Among them, hsa-miR-374a-5p and hsa-miR-374b-5p consistently rated highly across all computational approaches, indicating that these two miRNAs with the highest scores may be pivotal in the onset and progression of rectosigmoid junction cancer (Zhang et al., 2021b). These results suggest that the lncRNA-miRNA-mRNA network provides several molecules that may serve as novel prognostic biomarkers and therapeutic targets.

## 6 Engineered macrophage-targeted tumour therapy

Deciphering the intricate roles of macrophages in the tumor microenvironment and exploring methods to convert this understanding into clinical applications have been the main goals of recent research on macrophages in cancer immunotherapy. Numerous investigations have demonstrated that engineered immune cells—this emerging cellular immunotherapy—utilize genetically reprogrammed immune effectors engineered for pathological signal detection and targeted elimination (Weber et al., 2020). These immune cells can function as “living drugs” to stop the growth of tumor cells when given to patients. As a novel therapeutic approach, engineered macrophages exhibit a lot of promise, particularly in the areas of immunomodulation and targeted drug delivery.

Chimeric antigen receptor-modified macrophages (CAR-Ms), in which specific chimeric antigen receptors are genetically engineered into macrophages to acquire the ability to recognise against specific antigens, have also been reported to be in clinical use (Yang et al., 2024b). CAR-Ms express pro-inflammatory cytokines and chemokines, convert bystander M2 macrophages to M1, upregulate antigen-presentation mechanisms, recruit and deliver antigens to T cells, and resist the effects of immunosuppressive cytokines (Klichinsky et al., 2020). Michael Klichinsky et al. demonstrated that CAR-Ms also elicited a pro-inflammatory tumor microenvironment and augmented anti-tumor T-cell activity in a humanized mouse model (Klichinsky et al., 2020). Moreover, Paco López-Cuevas et al. demonstrated that the phagocytic uptake of artificial progenitor cell macrophages, which were loaded with anti-miR-223, by human macrophages significantly extended their pro-inflammatory state by obstructing the suppression of pro-inflammatory cytokines. This modification subsequently altered immune cell-cancer cell interactions and diminished tumor size by decreasing cell proliferation and enhancing cell death, ultimately resulting in a reduction of cancer burden (López-Cuevas et al., 2022). Macrophages have garnered significant interest as targets for medication delivery. Gao et al. created a virus-mimicking membrane-coated nucleic acid nanogel, designated as Vir-Gel, which incorporates miR-155. miR-155 attenuates the expression of TNF-α protein in macrophages, thereby enhancing the synthesis of pro-inflammatory cytokines and other mediators associated with the M1 phenotype. This reprograms macrophages from the pro-invasive M2 phenotype to the anti-tumor M1 phenotype, therefore limiting tumor cell proliferation (Gao et al., 2021). Thomas Kerzel’s team delineated a lentiviral vector platform that specifically modifies hepatic macrophages, encompassing Kupffer cells and TAMs, to provide type I interferon, IFNα, to liver metastases. The gene-based administration of IFNα inhibited the proliferation of liver metastases originating from colorectal and pancreatic ductal adenocarcinoma in murine models (Kerzel et al., 2023).

Although engineered macrophage therapy shows significant potential for antitumor treatment, its clinical application still faces multiple key challenges and limitations. In terms of safety, the use of macrophages to deliver drugs may induce unpredictable immune responses, including overactivation of the immune system and related risks, such as inflammatory storms or autoimmune-like symptoms (Yang et al., 2024b). Concerning stability, whereas *in vitro* produced M2-type macrophages provide therapeutic potential for tissue regeneration, their phenotypic stability is compromised by the chronic inflammatory microenvironment—ongoing pro-inflammatory signals may induce a reversion of polarization from M2 to M1 phenotype. Consequently, it is essential to thoroughly comprehend the mechanisms of macrophage polarization and the methods to augment the preferred phenotype via *in vitro* pretreatment (Na et al., 2023). In addition, further research is needed to improve the targeting of TAM and reduce non-specific macrophage interactions. Interactions with healthy macrophages result in high levels of off-target effects, which are a major obstacle to TAM therapy (Sylvestre et al., 2020).

As one of the research hotspots in the field of biomedicine, engineered macrophages have broad application prospects and important research value. In the future, with the continuous development and improvement of genetic engineering, cell engineering and other technologies, the research on engineered macrophages will be more in-depth and extensive.

## 7 Future and prospects

Macrophages have great potential as a source of cellular therapy, and their excellent regenerative ability can directly assist in the reconstruction and repair of tissues. At the same time, their powerful phagocytosis not only effectively removes cancer cells from the body but also protects against infectious agents. It is worth mentioning that certain drugs possess the potential to modulate macrophage polarization, providing new ideas and possibilities for intervening in macrophage function and thus regulating the tumor microenvironment by pharmacological means (Fernandez et al., 2022; Chen et al., 2023b). Increased emphasis has been directed towards the regulatory function of miRNAs in macrophage activation, polarization processes, tissue infiltration, and inflammation reduction. miRNAs display varied activities in the pathomechanisms of numerous diseases, indicating their potential as biomarkers for treatment and novel targets for therapeutic intervention. However, the same miRNA may play different or even opposite roles in different pathological processes. As an example, miR-125b, an antiviral host factor that restricts replication of porcine reproductive and respiratory syndrome virus, exerts its antiviral effects by negatively regulating cellular NF-κB signalling (Wang et al., 2024d). And Hannah M. Nelson et al. showed that elevated levels of miR-125b enhance three-dimensional growth and invasiveness of CRC and glioblastoma cell lines (Nelson et al., 2024). Consequently, to get a more comprehensive understanding of illness pathophysiology and to identify more efficient therapeutic methods, it is imperative to investigate the role of miRNAs in the progression of various diseases in more detail.

Colorectal cancer presents a significant barrier to clinical therapy owing to its considerable heterogeneity, medication resistance, and metastatic potential. Immunotherapy, as a novel therapeutic approach, provides new treatment options for patients with colorectal cancer. Engineered macrophages have garnered significant interest among many immunotherapeutic approaches due to their potential applications in the biomedical area; nonetheless, numerous problems remain to be addressed in their research and implementation. This encompasses guaranteeing the safety and usefulness of these cells, meticulously managing their function and activity, and investigating their applicability in clinical therapy, all of which necessitate comprehensive research and inquiry.

## Glossary

CRC: Colorectal cancer
miRNA: MicroRNAs
YO-CRC: young-onset CRC
TME: tumour microenvironment
TAM: tumour-associated macrophages
IARC: International Agency for Research on Cancer
IBD: inflammatory bowel disease
Stat3: Signal Transducer and Activator of Transcription 3
YAP: Yes-associated protein
UC: ulcerative colitis
DSS: dextran sulfate sodium
ARS2: arsenic resistance protein 2
C/EBPα: CCAAT enhancer binding protein α
TLR: Toll-like receptors
MyD88: myeloid differentiation primary response 88
HCC: hepatocellular carcinoma
IRAK1: interleukin-1 receptor-associated kinase 1
CVB3: coxsackievirus B3
EVs: Extracellular vesicles
LSS-EVs: laminar shear stress
IRE1: inositol-requiring enzyme 1
XBP1: X-box binding protein 1
GBM: glioblastoma
AR: allergic rhinitis
hnRNPA2B1: heterogeneous nuclear ribonucleoprotein A2B1
ROS: reactive oxygen species
EMT: epithelial-mesenchymal transition
CTCs: circulating tumor cells
**VEGF**vascular endothelial growth: vascular endothelial growthfactor
GABA: γ-aminobutyric acid
CAR-Ms: Chimeric antigen receptor-modified macrophages
HIF-1α: hypoxia-inducible factor-1α
IL-8: interleukin-8
PD-L1: programmed death ligand-1.
